# HLA-DQ2 and -DQ8 haplotypes frequency and diagnostic utility in celiac disease patients of Gaza strip, Palestine

**DOI:** 10.1007/s13317-017-0099-0

**Published:** 2017-11-15

**Authors:** Basim M. Ayesh, Eman Kh. Zaqout, Maged M. Yassin

**Affiliations:** 1 Department of Laboratory Medical Sciences, Alaqsa University, P.O. Box 4051, Gaza, Palestine; 2 Forensic Lab, Palestinian Ministry of Justice, Gaza, Palestine; 3 Faculty of Medicine, The Islamic University of Gaza, Gaza, Palestine

**Keywords:** Celiac disease, HLA-DQ2, HLA-DQ8, Gaza strip

## Abstract

**Purpose:**

Celiac disease (CD) diagnosis can be established by serological and small bowel biopsy (SBB), while absence of HLA-DQ2 and -DQ8 haplotypes excludes the disease. The present study aims at evaluating the diagnosis of a representative sample of pediatric and adult CD patients of Gaza strip in light of DQ2 and DQ8 haplotypes expression.

**Methods:**

Unrelated CD patients (*n* = 101) and matched healthy controls (*n* = 97) were genotyped for DQA1*05, DQB1*02 and DQB1*03:02 alleles by allele-specific real-time PCR. The diagnosis was re-evaluated according to the patient laboratory tests and HLA-DQ genotype.

**Results:**

The diagnosis of 35 patients who have been managed for CD could not be confirmed. Twenty-five of them were diagnosed upon their clinical presentation only. The remaining were either negative for serological and SBB tests or negative for HLA-DQ haplotypes. The HLA-DQ alleles were negative in 4 SBB and one Anti-EMA positive patients. The frequency of DQ2 and DQ8 haplotypes among the remaining 65 confirmed cases was 70.8 and 15.4%, respectively, compared to 17.5 and 27.8% in the controls. The DQB1*02 allele was the most common in the cases (84.6%) followed by DQA1*05 allele (80%) and DQB1*03:02 allele (20%). The DQA1*05 allele was commonest in the control group (54.6%) followed by DQB1*02 allele (42.3%) and DQB1*03:02 allele (28.9%).

**Conclusions:**

Absence of HLA-DQ2 and HLA-DQ8 genotyping in the workup of patients may result in CD misdiagnosis, particularly in a setting with poor histopathological diagnostic capacity.

## Introduction

Celiac disease (CD) is a chronic small intestinal immune-mediated enteropathy triggered by ingestion of a complex protein, gluten, present in cereals such as wheat, barley and rye in genetically predisposed individuals [1]. The most important determinant of genetic susceptibility for CD is the presence of human leukocyte antigen-DQ (HLA-DQ) heterodimers DQ2 (encoded by alleles DQA1*0501 and DQB1*0201) and HLA-DQ8 (encoded by alleles DQA1*0301 and DQB1*0302) [2–4]. HLA-DQ2 is present in more than 90% and HLA-DQ8 in about 5% of patients with CD [2, 5–7].

Expression of HLA-DQ2 or HLA-DQ8 molecules is necessary but not sufficient to cause CD. While about 20–40% of the white population carries HLA-DQ2, only 1% may develop the disease [7, 8]. Accordingly, addition of HLA-DQ typing to serological tests (anti-tissue transglutaminase enzyme antibodies tTG and Anti-endomysial antibodies EMA) does not improve the diagnostic capacity, because of its low positive predictive value [8, 9]. On the other hand, individuals lacking HLA-DQ2 and -DQ8 expression are unlikely to have CD with a negative predictive value of > 99% [4, 8, 9]. Therefore, the main role of HLA-DQ typing in the diagnosis of CD is to exclude the disease [6, 10, 11]. In particular, application of HLA-DQ2 and -DQ8 typing is recommended to exclude CD in patients with equivocal small bowel biopsy (SBB) finding or those following a Gluten-free diet [7, 12, 13] and patients with non-celiac gluten sensitivity [1, 14, 15]. HLA-DQ2 and -DQ8 typing is also recommended for CD screening in high-risk individuals including first-degree relatives of a confirmed case and patients with autoimmune and non-autoimmune conditions known to be associated with CD, such as type 1 diabetes mellitus, Down syndrome, and Turner syndrome [7, 16].

In Gaza strip, a total of 594 CD patients were registered and managed at Ard El Insan Palestinian Benevolent Association at the time this study was carried out. Their diagnosis did not include HLADQ genotyping and did not follow a standard protocol. To the best of our knowledge, only one published study discussed the immunodiagnosis of pediatric CD patients in Gaza strip [17]. Therefore, the aim of the present study was to evaluate the diagnosis of a representative sample of pediatric and adult CD patients of Gaza strip in light of DQ2 and DQ8 haplotypes.

## Materials and methods

### Study design and population

The study is a case–control study of 101 unrelated CD pediatric and adult patients (31 males and 70 females; mean age ± SD is 22.9 ± 15.8 years) and 97 healthy subjects, matched in age and sex (27 males and 70 females; mean age ± SD is 22.9 ± 15.8 years). All cases were collected from two centers of Ard El Insan Palestinian Benevolent Association in Gaza and Khan-Yunis governorates. The patients listed in the two centers were subdivided into three age groups from which representative numbers of patients were randomly recruited (50 were < 20 years; 35 were 20–40 years; 16 were > 40 years). The control group comprised unrelated healthy subjects with no symptoms of CD or gastrointestinal tract complains, recruited from the general population. Gender and age of controls was matched to the patients (31 males and 70 females; mean age ± SD is 23.5 ± 15.7 years).

### Sample collection and genomic DNA Extraction

About 3 ml of venous EDTA-anticoagulated blood were drawn and stored at 2–8 °C until genomic DNA extraction. DNA was extracted, within 3 days, from 300 µl whole blood samples using the Wizard Genomic DNA Purification kit (Promega, USA) according to the manufacturer’s instructions. The concentrations of DNA samples were determined spectrophotometrically, and adjusted to 15 ng/μL prior to the PCR reactions.

### HLA DQ2 and DQ8 genotyping

Sequence-specific primers for the DQA1*05, DQB1*02, DQB1*03:02 alleles and the Human Growth Hormone (HGH) endogenous control were previously described [18]. Separate 20 μl-amplification reactions were setup for each allele together with the HGH endogenous control. The reactions comprised 45 ng DNA, 1X EVA-Green Master Mix (Qiagen, Germany) and the proper primers concentration (Table 1). The amplification reactions were performed in the Rotor-Gene Q real-time PCR system (Qiagene, Germany) using a touchdown temperature profile (Table 2). The allele specific amplicons were identified with high resolution melting analysis (HRM). Results produced by the real-time PCR were called positive or negative based on the presence of the correct *T*_m_ (Table 3). The specificity of real-time PCR assay was further verified by loading some of the positive samples onto 2% agarose gel, stained with ethidium bromide and visualized using a UV-gel documentation system to rule out nonspecific amplification.

**Table 1:** Final concentrations of primers used in allele specific PCRs

	Primer Conc. (μM)		
	DQA1*05	DQB1*02	DQB1*03:02
AS primer	0.6	0.3	0.45
IC primer	0.3	0.3	0.21

*AS* allele specific, *IC* internal control

**Table 2:** Temperature profile for amplification and high resolution melting analysis of DQA1*05, DQB1*02 and DQB1*03:02 alleles

Amplification program	DQA1*05			DQB1*02 and DQB1*03:02		
	Temp	Time	Cycles	Temp	Time	Cycles
Hold	95 °C	5 min	1	95 °C	5 min	1
Amplification 1	95 °C 65 °C 72 °C	20 s 30 s 30 s	20	95 °C 68 °C 72 °C	20 s 30 s 30 s	10
Amplification 2	95 °C 60 °C 72 °C	20 s 30 s 30 s	20	95 °C 63 °C 72 °C	20 s 30 s 30 s	25
HRM	80–90 °C 0.1 °C increments for 2 s			82–90 °C 0.1 °C increments for 2 s

**Table 3:** T_m_ specific for amplicons of DQA1*05, DQB1*02 and DQB1*03:02 alleles and HGH internal control

Allele	*T*_m_ (°C)			
	Mean	Min.	Max.	SD
DQA1*05	82.4	81.6	83.2	0.3
DQB1*02	87.5	87.1	88.3	0.3
DQB1*03:02	87.7	87.1	88.5	0.3
HGH	85.8	85.2	86.6	0.3

### Data collection and analysis

Data was obtained from the patients’ medical records in Ard El Insan Palestinian Benevolent Association. The data focused on the results of laboratory tests performed to establish CD diagnosis (such as Anti-EMA, Anti-tTG and SBB).

Statistical analysis was carried out using the Statistical Package for Social Sciences (SPSS) for Windows, version 20. Chi-square test was applied to establish statistical significance with 95% confidence (*P* value ≤ 0.05).

## Results

### Clinical data and diagnostic tests

Table 4 summarizes the laboratory tests performed to establish diagnosis of the cases as registered in their medical records. The results of HLA typing test, performed in this study are also listed. All cases that were diagnosed by Anti-EMA and/or Anti-tTG in addition to SBB (nine cases) were positive for HLA-DQ2, -DQ8 haplotypes or one of their alleles. The diagnosis of 25 cases, surprisingly did not rely upon any laboratory test, but was solely based on their clinical presentation.

**Table 4:** Diagnostic test performed for the case group

Previous tests		HLA result^a^	
		Positive	Negative
Frequency (%)			
Anti-EMA			
Yes			
Positive	41 (40.6%)	40 (97.6%)	1 (2.4%)
Negative	6 (5.9%)	6 (100%)	0
No	54 (53.5%)	54 (100%)	0
Anti-tTG			
Yes			
Positive	7 (6.9%)	7 (100%)	0
Negative	1 (1%)	1 (100%)	0
No	93 (92.1%)	93 (100%)	0
Biopsy			
Yes			
Positive	31 (30.7%)	27 (87.1%)	4 (12.9%)
Negative	5 (5%)	5 (100%)	0
No	65 (64.4%)	65 (100%)	0
Anti-EMA and/or Anti-tTG + Biopsy			
Yes	9 (8.9%)	9 (100%)	0
No	92 (91.1%)	92 (100%)	0
Non^*b*^	25 (24.8%)	22 (88%)	3 (12%)

^a^Positive for HLA DQA1*05, DQB1*02 or DQB1*03:02 alleles

^b^Patients were considered positive for the disease based on their clinical picture only

Eight patients had neither DQ2 nor DQ8 haplotypes. Their diagnosis is thus excluded, particularly as four of them were diagnosed by SBB alone, three patients were diagnosed by their clinical pictures and one patient was diagnosed by an Anti-EMA test (Table 4).

In total, the diagnosis of 36 cases could not be confirmed because they lack any laboratory test, they were negative for all diagnostic tests, or they had a negative HLA typing test. Hence, the frequency of DQ2 and DQ8 haplotypes and their associated alleles was studied in the remaining 65 patients who were positive for a serological test and/or SBB and were positive for DQ2 and/or DQ8 haplotypes or any of their alleles.

### Distribution of DQA1*05, DQB1*02 and DQB1*03:02 alleles among confirmed patients and controls

The DQB1*02 allele was the most frequently encountered in confirmed CD patients (84.6%) followed by DQA1*05 allele (80%) and DQB1*03:02 allele (20%). On the other hand, DQA1*05 allele was the most frequently encountered in control individuals (54.6%) followed by DQB1*02 allele (42.3%) and last by DQB1*03:02 allele (28.9%). The difference in frequency of DQA1*05 and DQB1*02 between confirmed patients and controls was statistically significant (*P* = 0.001 and *P* = 0.000, respectively), while it was not significant for DQB1*03:02 allele (*P* = 0.203; Table 5).

**Table 5:** DQA1*05, DQB1*02 and DQB1*03:02 alleles distribution in patients and controls

	Confirmed cases (*n* = 65)	Controls (*n* = 97)	*P* value
DQA1*05 allele			
Positive	52 (80%)	53 (54.6%)	0.002
Negative	13 (20%)	44 (45.4%)	
DQB1*02 allele			
Positive	55 (84.6%)	41 (42.3%)	0.000
Negative	10 (15.4%)	56 (57.7%)	
DQB1*03:02 allele			
Positive	13 (20%)	28 (28.9%)	0.185
Negative	52 (80%)	69 (71.1%)	

Assuming Hardy–Weinberg equilibrium, allele frequencies were estimated from the percentage of individuals that carry the allele, using the Hardy–Weinberg equation (*p* ^*2*^ + *2pq* + *q* ^*2*^ = *1*). The estimated frequencies, in healthy controls, are (32.6%) for DQA1*05, (24.0%) for DQB1*02 and (15.7%) for DQB1*03:02.

### Distribution of DQ2 and DQ8 haplotypes among confirmed patients and controls

The DQ2 haplotype is more frequent in CD patients (70.8%) than in controls (17.5%), while the DQ8 haplotype is more frequent in controls (27.8%) than in CD patients (15.4%). Statistical analysis for the distribution of DQ2 haplotype among patients and controls reveals that the frequency difference is statistically significant (*P* = 0.000). The distribution is not statistically significant for DQ8 haplotype (*P* = 0.064) and for coexisting DQ2 and DQ8 haplotypes (*P* = 0.615; Table 6).

**Table 6:** Frequency of DQ2 and DQ8 haplotype among patients and controls

Haplotype	Confirmed cases *n* = (65)	Controls *n* = (97)	*P* value
DQ2			
Positive	46 (70.8%)	17 (17.5%)	0.000
Negative	19 (29.2%)	80 (82.5%)	
DQ8			
Positive	10 (15.4%)	27 (27.8%)	0.064
Negative	55 (84.6%)	70 (72.2%)	
DQ2 + DQ8			
Positive	3 (4.6%)	3 (3.1%)	0.615
Negative	62 (95.4%)	94 (96.9%)	

## Discussion

The diagnostic approach for CD in Gaza strip is based solely on serological or SBB tests. This approach does not meet the criteria of CD diagnosis published in different international guidelines such as NASPGHAN, ESPGHAN and BSG [1, 7, 19]. Based on these guidelines, a combination of gluten-dependent clinical manifestations, CD-specific antibodies, HLA-DQ2 and DQ8 haplotypes and enteropathy should be included [4, 7, 11, 20]. According to these guidelines only nine patients (8.9%) were complying with the diagnostic criteria. Therefore, in our study, we only included patients who were positive for serological test or intestinal biopsy and were positive for DQ2 or DQ8 haplotypes or for a fraction of the DQ2 heterodimer (DQA1*05 or DQB1*02 allele) for the rest of statistical analyses. The patients who fulfilled these criteria were only 65 patients and were considered confirmed for having the disease.

Typing for HLA-DQ2 and DQ8 is considered a useful tool to exclude CD or to make the diagnosis unlikely in the case of a negative test result for both markers. Therefore, ruling out the expression of DQA1*05, DQB1*02 and DQB1*03:02 is of critical importance for the exclusion of CD in suspected patients. In the current study eight patients (7.9%) were negative for the three tested alleles, accordingly they were excluded from being CD patients and they may need an extra investigation to other diseases such as wheat sensitivity and non-coeliac gluten sensitivity. Non-coeliac gluten sensitivity is poorly characterized and still debated if it is even caused by gluten at all, as opposed to other components of wheat (i.e., amylase–trypsin inhibitors, fermentable oligo-, di-, mono-saccharides and polyols) [21, 22].

Positive serological tests and SBB are essential to the diagnosis of CD when the patient is on a gluten-containing diet [1, 19]. In the current study, however, diagnosis of 2.4% of the Anti-EMA-positive (1/40), 12.9% of the biopsy-positive (4/27) and 12% of those diagnosed upon the clinical picture (3/22) was ruled out (Table 4). This result questions the reliability of SBB alone to establish CD diagnosis in Gaza strip, probably due to lack of the proper expertise. Accordingly, serological and particularly genetic tests would be much-admired to establish more accurate CD diagnosis in similar settings with poor histopathological diagnostic capacity. This is consistent with previous studies which predicted incorrect diagnosis in absence of genetic predisposing alleles with biopsy confirmed cases [23, 24]. Indeed, a recent large retrospective multicentre study conducted in Spain concluded that small bowel biopsy could have been avoided in a high proportion of symptomatic patients without any risk of misdiagnosis [13]. As a result, recent revisions of the criteria for CD diagnosis have addressed the increased contribution of HLA-DQ genotyping in addition to serology in CD diagnosis, especially for its high negative predictive value [4, 25, 26].

The HLA-DQ2 and -DQ8 were, respectively, present in 70.8% and in 15.4% of CD patients in Gaza Strip compared to 17.5 and 27.8% in controls, respectively. Our results differ from those described in European populations, where the frequency of the HLA-DQ2 is higher than 90% and that of HLA-DQ8 ranges between 5 and 10% [27, 28]. The frequency of DQ2 haplotype in controls (17.5%) is consistent with other Arab populations (9.6–16.4%) and European populations (14.1–9.6%); and the frequency of DQ8 (27.8%) is higher than in Arabs (10.2–14.3%) and Europeans (7.3–14.5%) as reported in the Allele Frequency Net Database [29].

Another finding from the present study was that 6 (9.2%) of the 65 coeliac patients expressed neither the DQ2 nor DQ8 heterodimers. Three of them expressed the DQA1*05 allele and the remaining three expressed the DQB1*02 allele. Several previous studies have demonstrated that just one of the alleles DQA1*05 or DQB1*02, each coding for half of the DQ2 heterodimer molecule would confer a predisposition towards triggering CD [27, 30–32].

Assuming Hardy–Weinberg equilibrium, the allelic frequency could be calculated from the percentage of control individuals who possess the allele and compared to published frequencies for other populations in the Allele Frequency Net Database [29]. The calculated frequencies (32.6% for DQA1*05, 24.0% for DQB1*02 and 15.7% for DQB1*03:02) came in the middle compared to other Arabs (13–37% for DQA1*05, 0–36% for DQB1*02 and 0–20.7% for DQB1*03:02). Our frequencies are slightly different from the general population of North America (0–30% for DQA1*05, 0–24% for DQB1*02 and 9.6–77.8% for DQB1*03:02) and Europe (0–40.8% for DQA1*05, 9–48% for DQB1*02 and 1.9–22% for DQB1*03:02). Any discrepancy in the frequency of alleles between our population and other populations may be attributed to a number of factors including: ethnicity and reproductive attitudes (i.e., consanguinity).

## Conclusions

Absence of HLA-DQ2 and HLA-DQ8 genotyping may result in CD misdiagnosis, particularly in a setting with poor histopathological diagnostic capacity.

In Gaza CD patients, the DQB1*02 is the most commonly encountered allele (84.6%) followed by DQA1*05 (80%) and DQB1*03:02 (20%). The DQA1*05 allele is the most commonly encountered in healthy control individuals (54.6%) followed by DQB1*02 (42.3%) and DQB1*03:02 (28.9%).

The HLA-DQ2 haplotype frequency is higher in patients (70.8%) than in controls (17.5%), while HLA-DQ8 is higher in controls (27.8%) than patients (15.4%).

## References

[1] Ludvigsson JF, Leffler DA, Bai JC, Biagi F, Fasano A, Green PH, Hadjivassiliou M, Kaukinen K, Kelly CP, Leonard JN, Lundin KE, Murray JA, Sanders DS, Walker MM, Zingone F, Ciacci C (2012). The Oslo definitions for coeliac disease and related terms. Gut.

[2] Megiorni F, Pizzuti A (2012). HLA-DQA1 and HLA-DQB1 in Celiac disease predisposition: practical implications of the HLA molecular typing. J Biomed Sci.

[3] Kim CY, Quarsten H, Bergseng E, Khosla C, Sollid LM (2004). Structural basis for HLA-DQ2-mediated presentation of gluten epitopes in celiac disease. Proc Natl Acad Sci USA.

[4] Husby S, Koletzko S, Korponay-Szabo I, Mearin M, Phillips A, Shamir R, Troncone R, Giersiepen K, Branski D, Catassi C (2012). European Society for pediatric gastroenterology, hepatology, and nutrition guidelines for the diagnosis of coeliac disease. J Pediatr Gastroenterol Nutr.

[5] Romanos J, van Diemen CC, Nolte IM, Trynka G, Zhernakova A, Fu J, Bardella MT, Barisani D, McManus R, van Heel DA, Wijmenga C (2009). Analysis of HLA and non-HLA alleles can identify individuals at high risk for celiac disease. Gastroenterology.

[6] Karell K, Louka AS, Moodie SJ, Ascher H, Clot F, Greco L, Ciclitira PJ, Sollid LM, Partanen J (2003). HLA types in celiac disease patients not carrying the DQA1*05-DQB1*02 (DQ2) heterodimer: results from the European Genetics Cluster on Celiac Disease. Hum Immunol.

[7] Husby S, Koletzko S, Korponay-Szabo IR, Mearin ML, Phillips A, Shamir R, Troncone R, Giersiepen K, Branski D, Catassi C, Lelgeman M, Maki M, Ribes-Koninckx C, Ventura A, Zimmer KP (2012). European Society for pediatric gastroenterology, hepatology, and nutrition guidelines for the diagnosis of coeliac disease. J Pediatr Gastroenterol Nutr.

[8] Wolters VM, Wijmenga C (2008). Genetic background of celiac disease and its clinical implications. Am J Gastroenterol.

[9] Hadithi M, von Blomberg BM, Crusius JB, Bloemena E, Kostense PJ, Meijer JW, Mulder CJ, Stehouwer CD, Pena AS (2007). Accuracy of serologic tests and HLA-DQ typing for diagnosing celiac disease. Ann Intern Med.

[10] Rubio-Tapia A, Hill ID, Kelly CP, Calderwood AH, Murray JA (2013). ACG clinical guidelines: diagnosis and management of celiac disease. Am J Gastroenterol.

[11] Ludvigsson JF, Bai JC, Biagi F, Card TR, Ciacci C, Ciclitira PJ, Green PH, Hadjivassiliou M, Holdoway A, van Heel DA (2014). Diagnosis and management of adult coeliac disease: guidelines from the British Society of Gastroenterology. Gut.

[12] Kaukinen K, Partanen J, Maki M, Collin P (2002). HLA-DQ typing in the diagnosis of celiac disease. Am J Gastroenterol.

[13] Donat E, Ramos JM, Sanchez-Valverde F, Moreno A, Martinez MJ, Leis R, Pena-Quintana L, Castillejo G, Fernandez S, Garcia Z, Ortigosa L, Balmaseda E, Marugan JM, Eizaguirre FJ, Lorenzo H, Barrio J, Ribes-Koninckx C (2015). ESPGHAN 2012 guidelines for coeliac disease diagnosis: validation through a retrospective Spanish multicentric study. J Pediatr Gastroenterol Nutr.

[14] Biesiekierski JR, Newnham ED, Irving PM, Barrett JS, Haines M, Doecke JD, Shepherd SJ, Muir JG, Gibson PR (2011). Gluten causes gastrointestinal symptoms in subjects without celiac disease: a double-blind randomized placebo-controlled trial. Am J Gastroenterol.

[15] Lundin KE, Alaedini A (2012). Non-celiac gluten sensitivity. Gastrointest Endosc Clin N Am.

[16] Ludvigsson JF, Bai JC, Biagi F, Card TR, Ciacci C, Ciclitira PJ, Green PH, Hadjivassiliou M, Holdoway A, van Heel DA, Kaukinen K, Leffler DA, Leonard JN, Lundin KE, McGough N, Davidson M, Murray JA, Swift GL, Walker MM, Zingone F, Sanders DS (2014). Diagnosis and management of adult coeliac disease: guidelines from the British Society of Gastroenterology. Gut.

[17] Ruby AM, Al-Khodary R, Shubair M, Sirdah M (2014). Immunodiagnosis of celiac disease among children with chronic diarrhea in Gaza Strip, Palestine. Am J BioSci.

[18] Profaizer T, Eckels D, Delgado J (2011). Celiac disease and HLA typing using real-time PCR with melting curve analysis. Tissue Antigens.

[19] Hill ID, Dirks MH, Liptak GS, Colletti RB, Fasano A, Guandalini S, Hoffenberg EJ, Horvath K, Murray JA, Pivor M, Seidman EG (2005). Guideline for the diagnosis and treatment of celiac disease in children: recommendations of the North American Society for Pediatric Gastroenterology, Hepatology and Nutrition. J Pediatr Gastroenterol Nutr.

[20] Hill ID, Dirks MH, Liptak GS, Colletti RB, Fasano A, Guandalini S, Hoffenberg EJ, Horvath K, Murray JA, Pivor M (2005). Guideline for the diagnosis and treatment of celiac disease in children: recommendations of the North American Society for Pediatric Gastroenterology, Hepatology and Nutrition. J Pediatr Gastroenterol Nutr.

[21] Henggeler JC, Veríssimo M, Ramos F (2017). Non-coeliac gluten sensitivity: a review of the literature. Trends Food Sci Technol.

[22] Volta U, Caio G, Karunaratne TB, Alaedini A, De Giorgio R (2017). Non-coeliac gluten/wheat sensitivity: advances in knowledge and relevant questions. Expert Rev Gastroenterol Hepatol.

[23] Kapitany A, Toth L, Tumpek J, Sipos E, Woolley N, Partanen J, Szegedi G, OlÁh É, Sipka S, Korponay-szabÓ I (2006). Diagnostic significance of HLA-DQ typing in patients with previous coeliac disease diagnosis based on histology alone. Aliment Pharmacol Ther.

[24] Anderson RP, Henry MJ, Taylor R, Duncan EL, Danoy P, Costa MJ, Addison K, Tye-Din JA, Kotowicz MA, Knight RE (2013). A novel serogenetic approach determines the community prevalence of celiac disease and informs improved diagnostic pathways. BMC Med.

[25] Hadithi M, Von Blomberg BME, Crusius JBA, Bloemena E, Kostense PJ, Meijer JW, Mulder CJ, Stehouwer CD (2007). Accuracy of serologic tests and HLA-DQ typing for diagnosing celiac disease. Ann Intern Med.

[26] Kurppa K, Salminiemi J, Ukkola A, Saavalainen P, Löytynoja K, Laurila K, Collin P, Mäki M, Kaukinen K (2012). Utility of the new ESPGHAN criteria for the diagnosis of celiac disease in at-risk groups. J Pediatr Gastroenterol Nutr.

[27] Karell K, Louka AS, Moodie SJ, Ascher H, Clot F, Greco L, Ciclitira PJ, Sollid LM, Partanen J, Disease EGCoC (2003) HLA types in celiac disease patients not carrying the DQA1* 05-DQB1* 02 (DQ2) heterodimer: results from the European Genetics Cluster on Celiac Disease. Human Immunol 64(4):469–477 10.1016/s0198-8859(03)00027-2 12651074

[28] Tye-Din J, Anderson R (2008) Immunopathogenesis of celiac disease. Curr Gastroenterol Rep 10(5):458–465 10.1007/s11894-008-0085-9 18799120

[29] Gonzalez-Galarza FF, Takeshita LY, Santos EJ, Kempson F, Maia MH, da Silva AL, Teles e Silva AL, Ghattaoraya GS, Alfirevic A, Jones AR, Middleton D (2014) Allele frequency net 2015 update: new features for HLA epitopes, KIR and disease and HLA adverse drug reaction associations. Nucleic Acids Res 43(Database issue):D784–788. 10.1093/nar/gku1166 10.1093/nar/gku1166 PMC4383964 25414323

[30] Kagnoff MF (2007) Celiac disease: pathogenesis of a model immunogenetic disease. J Clin Investig 117(1):41–49 10.1172/JCI30253 PMC1716218 17200705

[31] Polvi A, Arranz E, Fernandez-Arquero M, Collin P, Maki M, Sanz A, Calvo C, Maluenda C, Westman P, de la Concha EG, Partanen J (1998) HLA-DQ2-negative celiac disease in Finland and Spain. Hum Immunol 59(3):169–175 10.1016/s0198-8859(98)00008-1 9548076

[32] Mubarak A, Spierings E, Wolters V, van Hoogstraten I, Kneepkens CF, Houwen R (2013) Human leukocyte antigen DQ2. 2 and celiac disease. J Pediatr Gastroenterol Nutr 56(4):428–430 10.1097/MPG.0b013e31827913f9 23085892

